# Challenges for Estimating the Global Prevalence of Micronutrient Deficiencies and Related Disease Burden: A Case Study of the Global Burden of Disease Study

**DOI:** 10.1093/cdn/nzab141

**Published:** 2021-11-18

**Authors:** Sonja Y Hess, Alexander C McLain, Edward A Frongillo, Ashkan Afshin, Nicholas J Kassebaum, Saskia J M Osendarp, Reed Atkin, Rahul Rawat, Kenneth H Brown

**Affiliations:** Institute for Global Nutrition and Department of Nutrition, University of California, Davis, Davis, CA, USA; Department of Epidemiology and Biostatistics, Arnold School of Public Health, University of South Carolina, Columbia, SC, USA; Department of Health Promotion, Education, and Behavior, Arnold School of Public Health, University of South Carolina, Columbia, SC, USA; Institute for Health Metrics and Evaluation, University of Washington, Seattle, WA, USA; Institute for Health Metrics and Evaluation, University of Washington, Seattle, WA, USA; The Micronutrient Forum, Washington, DC, USA; The Micronutrient Forum, Washington, DC, USA; Bill & Melinda Gates Foundation, Seattle, WA, USA; Institute for Global Nutrition and Department of Nutrition, University of California, Davis , Davis, CA, USA

**Keywords:** global burden of disease, iodine, iron, micronutrient, prevalence, vitamin A, zinc, deficiency

## Abstract

Information on the prevalence of micronutrient deficiencies is needed to determine related disease burden; underpin evidence-based advocacy; and design, deliver, and monitor safe, effective interventions. Assessing the global prevalence of deficiency requires a valid micronutrient status biomarker with an appropriate cutoff to define deficiency and relevant data from representative surveys across multiple locations and years. The Global Burden of Disease Study includes prevalence estimates for iodine, iron, zinc, and vitamin A deficiencies, for which recommended biomarkers and appropriate deficiency cutoffs exist. Because representative survey data are lacking, only retinol concentration is used to model vitamin A deficiency, and proxy indicators are used for the other micronutrients (goiter for iodine, hemoglobin for iron, and dietary food adequacy for zinc). Because of data limitations, complex statistical modeling is required to produce current estimates, relying on assumptions and proxies that likely understate the extent of micronutrient deficiencies and the consequent global health burden.

## Teaser

In this review, steps needed to estimate micronutrient deficiency prevalence and related disease burdens are described; challenges are outlined using the Global Burden of Disease Study as a case study.

## Introduction

Micronutrient deficiencies result in a broad range of adverse health consequences, including increased infectious disease, growth restriction, physical disabilities, and impaired neurocognitive development (1–4). According to the most recent Lancet Series on maternal and child undernutrition (5), maternal and child undernutrition, including anemia and deficiencies of zinc, vitamin A, and other micronutrients, remain major global health concerns. Despite the serious consequences of these deficiencies for the individual and for society, there are limited data on vitamin and mineral status of human populations from nationally representative surveys, especially in low- and middle-income countries. This lack of information hinders global, regional, and national efforts to prevent micronutrient deficiencies and their consequences (6, 7). Information on the prevalence of micronutrient deficiencies is needed to assess the related disease burden; underpin evidence-based advocacy; and design, deliver, target, and monitor safe, effective, and sustainable intervention programs (8–10). Moreover, information on the prevalence of anemia and micronutrients is needed to track progress toward the Global Nutrition Target 2 (50% reduction in the prevalence of anemia in women of reproductive age by the year 2025), Sustainable Developmental Goals (SDGs) indicator 2.2.3 (prevalence of anemia in women 15–49 y of age, by pregnancy status), and SDG 3 (good health and well-being) (11, 12).

The collection of population-level data through regular surveys, such as the Demographic Health Surveys (DHSs) supported by the United States Agency for International Development and representative surveys led by national governments, allows tracking of multiple sets of indicators (13). Data on anemia and micronutrient deficiencies from many nationally and subnationally representative surveys are available in the Vitamin and Mineral Nutrition Information System (VMNIS), an interactive data repository managed by the WHO (14). The WHO previously developed global estimates of vitamin A deficiency and anemia using this information (15–18). The Iodine Global Network aggregates and tracks iodine status through the Global Iodine Scorecard (19). The global risk of zinc deficiency was estimated by Wessells and Brown (20). Recent reviews have also attempted to describe the public health burden of thiamin, vitamin D, and folate deficiencies (21–23). As part of the Global Burden of Disease (GBD) Study, the prevalence and related disease burden of anemia are estimated, along with deficiencies of iodine, iron, zinc, and vitamin A. These estimates are updated every 1–2 y (24, 25).

The motivation for global health metrics, such as the Global Health Estimates by the WHO (26) and the GBD Study (24), is to provide policy makers with data on disease prevalence, trends over time, and the causes of and risk factors for death and disability. Global health metrics may shed light on public health problems that would otherwise be neglected (27). Specifically, the estimated prevalence of micronutrient deficiencies is used to guide funding agencies and policy makers to prioritize the allocation of global and national resources toward micronutrient programs or other public health and social development interventions, such as water, sanitation and hygiene, early childhood education, and malaria control and prevention, among others.

Considering that global health metrics influence investment priorities and may ultimately affect the health and well-being of the most vulnerable population groups, it is important to understand the underlying data and methods used to generate these statistics. Estimating the global disease burden due to any risk factor requires the integration of multiple types and sources of data and the use of statistical modeling to harmonize the data and impute data gaps for estimating prevalence. Combining these with other sources of information (e.g., the health impact of micronutrient deficiency) to generate measures of disease burden and accompanying summary measures further requires numerous interconnected assumptions. The objective of the present article is to review the data required and modeling methods and assumptions used to generate estimates of the prevalence and related disease burden of micronutrient deficiencies. The challenges that must be overcome are described, using the GBD Study as a case study. The underlying data and methods used for each of the 4 micronutrients in GBD are not the same. Thus, in the following sections, we summarize details about the data sources and methods used for each of the micronutrient deficiencies currently included in GBD. Dissemination of the methods is critical so that any limitations of the analyses can be understood and evaluated by experts in the field.

## Steps Required to Estimate the Prevalence of Micronutrient Deficiencies and Associated Disease Burden

A biomarker of micronutrient status is a measure or indicator of exposure, status, and function (28); more specifically, in the present review, a biomarker is defined as a measure or indicator present in body fluids or tissue. Sparse information on deficiency prevalence can be bolstered by data on related measures or indicators, although use of these indirect indicators of micronutrient status requires assumptions that may undermine the accuracy and precision of the estimate. For example, dietary intake data have been used to determine the prevalence of dietary inadequacy, which is then used as a surrogate for the prevalence of deficiency. In other cases, nutrient availability data, based on national food balance sheets (FBSs) or Supply Utilization Accounts (SUAs) prepared by the FAO, have been used to estimate the risk of dietary inadequacies. Dietary inadequacy, however, cannot be considered equivalent to micronutrient deficiency for several reasons. For example, some micronutrients may be consumed seasonally and stored in the body, so intermittent inadequacy of intake may not result in deficiency. Also, micronutrients may be consumed from unmeasured sources, such as supplements, ambient water, and soil contamination of foods, or produced by gut flora or fermentation of food. Estimating the dietary intake based on a single 24-h recall also has several weaknesses due to common measurement errors and within-person (day-to-day) variation in nutrient intake (29, 30). The use of food availability as a proxy is even more controversial, because FBS and SUA data reflect neither true dietary intakes nor the distribution of intakes across the population (31), so they would not be expected to correlate closely with deficiency prevalence based on biochemical data obtained from selected population subgroups (32). Thus, dietary data have fundamental weaknesses because they only indicate a possible risk of micronutrient deficiency, but do not provide direct information on micronutrient status (6).

**FIGURE 1:**
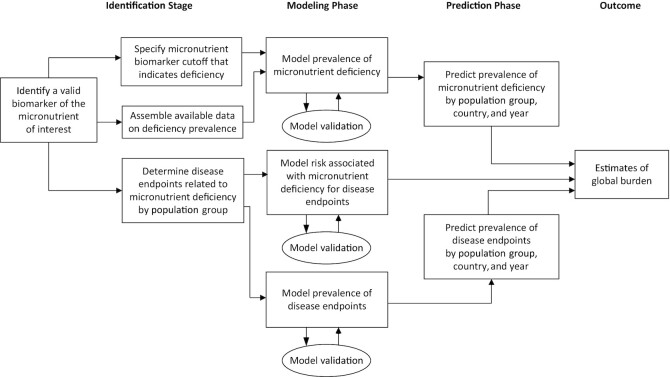
Steps in determining the global disease burden attributable to selected micronutrient deficiencies.

Several steps are required to develop estimates of the disease burden imposed by micronutrient deficiencies (**Figure 1**). First, information is needed on the prevalence of the deficiency, which in turn requires that *1*) a valid biomarker of the status of the micronutrient of interest has been identified; *2*) appropriate cutoffs have been agreed upon to define deficiency, based on either the threshold of the biomarker at which health impairments begin to occur, or a statistically defined cutoff; and *3*) data on the prevalence of deficiency are available across multiple locations and years from representative samples of the populations or population subgroups of interest. In addition, there is a need for adequate scientific evidence from diverse populations to link the micronutrient deficiency with adverse health outcomes and quantify the magnitude and strength of those relations. Lastly, to ensure full transparency, adequate documentation of assumptions and methods is needed for any modeling exercise producing global health estimates.

For each of the 4 micronutrients included in the GBD Study, ≥1 reliable biomarker has been recommended by expert groups (**Table 1**). Specifically, urinary iodine concentration is recommended as a marker of population iodine exposure (33–35), and more recently plasma or serum thyroglobulin has been proposed as an indicator of iodine status (35). If the prevalence of goiter is used as an indicator of iodine deficiency, the WHO recommends combining grade 1 goiter (i.e., goiter is palpable, but not visible) with grade 2 goiter (i.e., visible goiter) to calculate the total goiter prevalence (36). Plasma ferritin and soluble transferrin receptor concentrations are recommended for assessing iron status (37–40), plasma zinc concentration for zinc status (41–43), and plasma retinol or retinol-binding protein concentration for vitamin A status (44, 45). For each of these micronutrients, there is consensus on the cutoff to define deficiency (34, 38, 39, 45, 46), and concentrations of ferritin, soluble transferrin receptor, zinc, retinol, and retinol-binding protein may require adjustment for inflammation (47–50).

**TABLE 1:** Biomarkers recommended by expert groups to determine micronutrient deficiency at the population level^1^

Micronutrient	Recommended biomarker	Expert group	References
Iodine	Urinary iodine	BOND, IGN, WHO	(34, 35)
	s/p thyroglobulin	BOND	(35)
Iron	s/p ferritin	BOND, WHO	(37, 38, 40)
	s/p soluble transferrin receptor	BOND, WHO	(39, 40)
Zinc	s/p zinc	BOND, IZiNCG	(41–43)
Vitamin A	s/p retinol	BOND, WHO	(44, 45)
	s/p retinol-binding protein concentration	BOND	(44)

1 BOND, Biomarkers of Nutrition for Development; IGN, Iodine Global Network; IZiNCG, International Zinc Nutrition Consultative Group; s/p, serum or plasma.

Micronutrient status data available from different sources indicate variable coverage depending on the particular micronutrient. The WHO VMNIS is a curated, publicly accessible data repository on the hemoglobin and micronutrient status of representative population groups (14), although some national survey data are either not publicly available or not yet included in this database. Hemoglobin is the biomarker most frequently available in the VMNIS, with other micronutrient biomarkers reported less often (**Table 2**). For example, plasma zinc concentration was assessed among young children only in 35 surveys from 27 countries, and in 9 surveys among women of reproductive age. Similarly, there is limited information on micronutrient intakes from nationally or regionally representative dietary surveys. The Global Dietary Database 2017 identified a total of 1220 dietary surveys, but only 113 surveys assessed dietary iron intake, 78 surveys assessed dietary zinc, and <20 surveys each assessed vitamin A (including vitamin A supplement intake) and iodine intake (51). Thus, there is a scarcity of information on population micronutrient status based on reliable biomarkers and dietary intake (7), and there currently is inadequate information to estimate the global prevalence of iron and zinc deficiencies reliably for the years of interest from 1990 to the present, which are modeled in the GBD Study.

**TABLE 2:** Overview of representative surveys at the national and regional levels that determined hemoglobin and micronutrient status indicators included in the VMNIS of the WHO^1^

	Preschool children		School children		Women of reproductive age	
	Surveys, *n*	Countries, *n*	Surveys, *n*	Countries, *n*	Surveys, *n*	Countries, *n*
Hemoglobin	400	127	114	59	251	98
Urinary iodine concentration	13	11	197	110	34	27
Plasma ferritin	90	63	38	26	33	28
Transferrin receptor	27	19	9	7	8	8
Plasma zinc	35	27	19	14	9	9
Plasma retinol	112	75	33	25	22	20
Retinol-binding protein	28	22	5	4	9	7

1 For the VMNIS, see (14). Number of surveys implemented between 1990 and 2020 included in VMNIS as of 5 May, 2021. VMNIS, Vitamin Mineral Nutrition Information System.

After extracting all available and relevant data, the next step is standardization of data around a reference definition, then combining this information with demographic data and predictive covariates to statistically model the relations and extrapolate the prevalence to other locations (i.e., other countries), population subgroups, and time points for which data are not available. This step is commonly done with spatiotemporal modeling techniques, which use the available prevalence data and information on correlates to the particular micronutrient deficiency (e.g., macroeconomic indicators), along with the spatial and temporal relations among the data. These methods develop statistical relations between the available prevalence data and the predictors (e.g., correlates, space and time) which are then used to predict the unknown prevalence values. The rise in popularity of machine learning and complex modeling techniques in recent years (52) has led to the point where, for some, “black box” algorithms make it difficult to determine the impact of predictors and their consistency across countries and population subgroups. This has led to a push for explainable machine learning algorithms and emphasizes the underlying importance of clear documentation of the models (53).

The final step is to identify disease endpoints caused by the micronutrient deficiency and quantify the increased risk associated with having a particular micronutrient status. This is typically done using the hierarchy of scientific evidence (54), where published studies from intervention trials and cohort studies are the preferred sources of risk measurement. Cross-sectional case-control and observational studies can also be considered in certain circumstances, where risk of recall or measurement bias is low. All study findings need to be appropriately meta-analyzed—including controlling for confounding and quantification of residual heterogeneity—to determine the overall RR. These data can potentially be supplemented with additional information to quantify differential risk between subgroups.

Additional important modeling considerations are both estimation of robust uncertainty intervals and validation of these uncertainty intervals. For models with multiple stages, estimates of uncertainty need to propagate error from one stage to the next, which can be completed using bootstrapping techniques for frequentist approaches (55) or via Bayesian analyses with appropriately chosen prior distributions (56). Validation of uncertainty intervals and predictive estimates can be completed with out-of-sample predictive validity tests or (preferably) newly obtained empirical data.

## General Overview of the GBD Study

The GBD Study, which is led by the Institute for Health Metrics and Evaluation (IHME) at the University of Washington, is an international collaboration of >7500 researchers in 136 countries, herein referred to as the GBD Collaboration, who work to generate modeled estimates of comparative health loss from >300 diseases and injuries and 87 risk factors in 204 countries and territories, disaggregated by age and sex, from 1990 to the present, allowing comparisons over time, across age groups, and among populations (25). Thus, the effects of micronutrient deficiencies are just one of multiple analyses that are completed. The GBD Study catalogues all input data for each model in the Global Health Data Exchange, using data from the VMNIS whenever possible and supplementing with additional representative surveys, national reports, published studies, and other sources identified by GBD collaborators.

To estimate the disease burden of anemia and micronutrient deficiencies, the GBD Study uses 2 main methods: *1*) causal attribution methods, where specific micronutrient deficiencies are considered as an underlying cause of a particular disease burden (e.g., blindness *due to* vitamin A deficiency) (57); and *2*) the risk factor method, where the micronutrient deficiency is a risk factor for other diseases (e.g., diarrhea *attributable to* vitamin A deficiency as a risk factor) (58). The modeling strategy for these 2 distinct scenarios uses parallel processes that are internally consistent for each micronutrient, but the results are presented separately by GBD. For those micronutrients considered in the GBD Study as both causes of disease and risk factors for disease (namely, vitamin A deficiency and iron deficiency), the burden attributable to the risk factor, by definition, includes the burden due to the causal attribution. Thus, results of the 2 methods are not, and should not be, summed.

With both methods, the GBD Collaboration first estimates the frequency and burden of the condition within the population. A variety of data sources are consulted to estimate the epidemiology of each disease or injury. These data sources are, for example, inpatient and outpatient hospital records and health insurance claims, household surveys, micronutrient status surveys, published scientific studies, government reports, and results from cause-of-death models to inform estimates (59). Data are extracted, processed, standardized, and modeled to produce internally consistent estimates of prevalence and incidence. Separate sources of data on distribution of symptoms are then used to distribute prevalence cases into different sequelae classes, which serve as the units of calculation of years of life lived with disability (YLDs). For the causal attribution method, cause-specific deaths are considered along with global standard life expectancy to calculate years of life lost (YLLs) and prevalent cases are divided into different categories of severity, called sequelae, where the prevalence of each sequela is multiplied against a corresponding disability weight to calculate YLDs, accounting for comorbidity of diseases. The disability weight scales were determined from separate disability weight surveys (60, 61) and range from 0 (implying no loss of health) to 1 (implying health loss equivalent to death). The disability weights were found not to vary significantly by sex, location, or education status, so they are applied uniformly across geography, age, and time. Summing YLLs and YLDs results in estimates of disability-adjusted life years (DALYs). Micronutrients that are analyzed as GBD causes of specific diseases include iodine deficiency (for which disease sequelae include visible goiter and cretinism), dietary iron deficiency (sequelae include only anemia), and vitamin A deficiency (sequelae include asymptomatic vitamin A deficiency and vision impairment) (57).

For the GBD risk factor method, estimates of exposure are either expressed in terms of prevalence (same as GBD causes; e.g., anemic yes/no) or based on biomarker concentration (e.g., low hemoglobin concentration for iron deficiency risk exposure). The exposures are combined with meta-analyzed outcome-specific RRs to calculate population-attributable fractions (PAFs) and then to estimate the attributable deaths, YLDs, and DALYs due to specific risk factors for particular diseases. Micronutrient deficiencies that are analyzed as GBD risk factors include iron, zinc, and vitamin A deficiency (58).

Connecting and measuring the association of risk factors with disease outcomes is a key part of estimating global disease burden. In the GBD 2019 Study, the GBD Collaboration commonly performed meta-analyses using a procedure referred to as meta regression—Bayesian, regularized, trimmed (MR-BRT). MR-BRT is a Bayesian meta-regression model that allows for the inclusion of covariates to explain between-study bias and variability, and adds prior probability distributions, which can include any previous information on these parameters, to deal with data sparsity (62). The main features that set MR-BRT apart from other meta-analysis methods are *1*) relaxation of the log-linear assumption inherent in all meta-regressions by incorporation of flexible splines and *2*) implementation of likelihood-based designation of outlier data which allows for trimming of data from the model and prevents outlier studies from biasing the final result. The user specifies an outlier percentage; once this parameter is set the model and which data points are ignored (if any) are estimated simultaneously. The final trimming value is based on quantitative measures like model fit criteria or cross-validation, or more qualitative measures like identifying the lowest trim percentage that produces stable mean results. A methods article by Zheng et al. (62) describing MR-BRT includes an example of a meta-analysis of vitamin A supplementation on diarrheal disease using MR-BRT. The trimming results in 2 of the 12 studies being ignored, which changes the effect size from −0.15 without trimming to −0.05 with trimming. For GBD 2019, MR-BRT was applied with 10% trimming of the data. Exposure for each risk factor is then summarized and presented as a summary exposure value (SEV) to permit comparison across risk factors. SEV calculation combines measurement of exposure, size of impact (i.e., maximum RR), and attributable burden into a single number, but does not always easily translate into corresponding measures of biomarker-based micronutrient deficiency.

**Table 3** summarizes the micronutrient deficiency and related health burden results estimated in the GBD 2019 Study. In the following sections, we provide a more detailed overview of the GBD methods, and critiques from a nutritionist perspective, on estimating the disease burden imposed by deficiencies of iodine, iron, vitamin A, and zinc.

**TABLE 3:** The estimated prevalence (95% CI) of iodine, dietary iron, iron, zinc, and vitamin A deficiency and the associated global burden as estimated in the GBD Study for the year 2019^1^

	Iodine deficiency^2^	Dietary iron deficiency^3^	Iron deficiency^4^	Zinc deficiency^5^	Vitamin A deficiency^6^
Prevalence of deficiency					
Ages 1–4 y	0.0011 (0.0007, 0.0016)	0.29 (0.27, 0.30)	—	0.09 (0.03, 0.18)	0.16 (0.14, 0.17)
All ages	0.024 (0.019, 0.029)	0.14 (0.14, 0.15)	—	n/a	0.063 (0.061, 0.066)
Deaths (thousands) due to deficiency	—	—	42 (15, 70)	2.8 (0.7, 6.5)	24 (3, 50)
YLDs (thousands) due to deficiency	2439 (1373, 4239)	28,535 (19,128, 41,139)	28,798 (19,425, 41,492)	17 (5, 39)	1222 (833, 1711)
DALYs (thousands) due to deficiency	2439 (1373, 4239)	28,535 (19,128, 41,139)	31,263 (21,272, 43,987)	259 (67, 597)	3297 (1347, 5594)
SEV due to deficiency	—	—	19.57 (18.11, 21.12)	8.78 (2.89, 17.60)	15.01 (13.55, 16.86)
Total DALYs due to deficiency, %	0.10% (0.06%, 0.16%)	1.12% (0.80%, 1.51%)	1.20% (0.91%, 1.60%)	0.01% (0.003%, 0.02%)	0.13% (0.06%, 0.22%)

1 For the GBD Study 2019 see (57, 58). DALY, disability-adjusted life-year; GBD, Global Burden of Disease; n/a, not available; SEV, summary exposure value; YLD, year lived with disability.

2 Iodine deficiency modeled based on visible goiter.

3 Risk exposure for dietary iron deficiency modeled based on hemoglobin concentration below the anemia cutoff after accounting for other known anemia causes, and after accounting for known causes of iron deficiency (such as hookworm, schistosomiasis, upper gastrointestinal bleeding, and gynecologic conditions).

4 Risk exposure for iron deficiency modeled based on hemoglobin concentration below the anemia cutoff after accounting for other known anemia causes.

5 Zinc deficiency modeled based on dietary zinc inadequacy estimated from dietary surveys and FAO Supply Utilization Accounts. The GBD estimates for zinc deficiency are modeled for children aged 1–4 y only.

6 Vitamin A deficiency defined as serum retinol concentration < 70 μmol/L.

## Estimate of Disease Burden Due to Iodine Deficiency in the GBD 2019 Study, Using the Causal Attribution Method

### Estimate of prevalence of iodine deficiency

As noted, the estimate of the disease burden due to iodine deficiency only uses the causal attribution method, based on the understanding that iodine deficiency is a unique cause of goiter and cretinism. For the GBD 2019 Study, the estimates for the prevalence of visible goiter and cretinism were based on studies archived in the WHO VMNIS (14).

### Estimate of disease burden due to iodine deficiency

The modeling strategy for visible goiter due to iodine deficiency in GBD 2019 is a 2-step process (57). The initial model captures the age trend in the prevalence data, which is used to split the data into narrower age ranges. The prevalence of visible goiter is then modeled using the data disaggregated by age group using disease modeling—meta regression (DisMod-MR) 2.1, a hierarchical Bayesian spatial meta-analysis method that borrows strength across location and time to inform estimates where data are sparse or absent. Several new assumptions were introduced into this model in 2019 to allow for the possibilities that *1*) visible goiter incidence does not increase with age, *2*) a small amount of remission is possible, and *3*) the prevalence is 0 at birth. These assumptions were based on scientific evidence suggesting that the highest prevalence of visible goiter occurs among middle-age individuals and were prompted by observations that the previous, stricter parameters were limiting the predictive power of the model. The proportion of households using iodized salt and estimated sodium intake were included in the model as country-level covariates. No out-of-sample predictive validity testing was performed on DisMod-MR 2.1 models of goiter.

The prevalence of intellectual disability due to iodine deficiency is estimated by regressing the prevalence of cretinism (on the logit scale) on the prevalence of goiter (also logit transformed), based on studies reporting both conditions in the same population (57). This fitted model is then used to predict cretinism for all country locations using the goiter estimates from the DisMod-MR 2.1 model. Locations where the total goiter prevalence is <20% or household coverage of iodized salt is >90% were assumed to have 0 intellectual disability due to iodine deficiency. Data on intellectual disability due to iodine deficiency among children 5 y of age were used as incidence input in a second DisMod-MR 2.1 model to generate estimates for location-, year-, age-, and sex-specific population groups (57). Alongside incidence estimates, data from several published studies on the RR of mortality for people with intellectual disability are included in the model to capture the long-term mortality risk attributable to intellectual disability (63) and are used to estimate the relation between mortality risk and age. As a last step, the disabilities due to goiter and intellectual impairment are assigned based on disability weights that are constant regardless of age, gender, or location.

### Critique of methods used for iodine-related estimates

Whereas visible goiter and cretinism are the most severe manifestations of iodine deficiency, palpable nonvisible goiter is presently ignored in the GBD Study, and studies with goiter prevalence are outdated (64). Moreover, there is some evidence that mild to moderate iodine deficiency may impair children's cognitive development (65), even without visible goiter. This is not considered in the disease burden estimates, however, because there is insufficient scientific evidence from randomized controlled trials (66). Thus, the full health impact of iodine deficiency is likely underestimated in the GBD Study, and an important way forward will be to develop methods to estimate prevalence of iodine deficiency based on urinary iodide and to fully evaluate the evidence for iodine deficiency as a risk factor for other conditions.

## Estimates of Disease Burden Due to Iron Deficiency in the GBD 2019 Study

Both the causal attribution and risk factor modeling strategies are used in the GBD Study to reflect 2 different conceptual definitions of iron deficiency (57, 58). The causal attribution model is used to estimate the burden of “dietary iron deficiency,” which isolates the anemia-specific disease burden due just to inadequate dietary iron intake and not to the multiple other diseases, such as infections and blood loss, that can manifest as absolute or functional iron deficiency. In parallel, the risk factor model quantifies the aggregate exposure to iron deficiency, regardless of the underlying cause. Thus, iron deficiency as a risk factor is based on all subtypes of anemia for which iron deficiency (i.e., low intake, poor absorption, or excess loss) is theoretically a contributing factor and which thus have the potential to respond to iron supplementation (58). For both modeling methods, exposure to iron deficiency is determined based on the distribution of hemoglobin concentration, as will be explained. Although the 2 modeling methods are presented separately in GBD 2019, they are modeled together using an internally consistent framework. The burden attributable to the GBD 2019 risk factor method of iron deficiency also includes the burden identified with the causal attribution method.

### Estimate of disease burden due to dietary iron deficiency in the GBD 2019 Study, using the causal attribution method

#### Estimate of prevalence of dietary iron deficiency anemia

Dietary iron deficiency is determined from the GBD estimation of overall anemia, and therefore represents only iron deficiency associated with anemia and does not include iron deficiency without anemia. The anemia model has 2 main steps: *1*) estimation of overall anemia prevalence, and *2*) assignment of anemia to underlying causes (causal attribution). The GBD Collaboration estimated the anemia prevalence using 2 Spatio-Temporal Gaussian Process Regression (ST-GPR) models: 1 for mean hemoglobin concentration and 1 for SD of hemoglobin to estimate the center and spread of hemoglobin distribution. ST-GPR is a general modeling tool developed by the GBD Collaboration to generate estimates of population-level quantities smoothed over geography and time. It uses 3 steps starting with mixed-effects regression, followed by spatiotemporal weighting, and smoothing of residuals. New to GBD 2019 is the addition of ensemble model selection to the first stage of ST-GPR models, where a suite of candidate covariates were utilized in a train-test-test approach and ranked based on out-of-sample predictive validity (57). An ensemble of distribution families was then fit to individual-level data on hemoglobin concentration, again using a train-test approach where different combinations of distribution families were selected based on structured out-of-sample predictive validity testing using a combination of data from high-income and low- and middle-income countries. These distributions were combined with ST-GPR estimates of mean and SD of hemoglobin concentrations to estimate a full distribution of hemoglobin concentration for each location, year, age group, and sex. Prevalence estimates were derived using the WHO cutoff definitions for anemia (67), except for the <1-mo-old infants, for which there are no international recommendations available (68). The anemia prevalence for pregnant and nonpregnant females were modeled separately because of the different cutoffs, with a fixed estimate of the impact of pregnancy status based on an MR-BRT model evaluating the difference in hemoglobin between pregnant and nonpregnant persons as a function of age.

The second step of GBD anemia estimation is to assign each case of anemia to a specific cause. The inputs for this analysis are the previously determined distributions of hemoglobin; the prevalence of the different causes of anemia in each population, which range from malaria to schistosomiasis, menstrual disorders, kidney disease, hemoglobinopathies, and many more (**Supplemental Table 1**); and estimates of the hemoglobin shift for each cause of anemia (i.e., the average change in hemoglobin due to the cause). The hemoglobin distribution after accounting for the impact of a specific cause is obtained by shifting the hemoglobin distribution by the product of the prevalence of that cause and the hemoglobin shift of the cause. The remaining anemia after adjusting for all known causes is then defined as “residual anemia cases,” which are assigned to 5 causes: dietary iron deficiency; other infectious diseases; other neglected tropical diseases; other endocrine, nutrition, blood, and immune disorders; and other hemoglobinopathies and hemolytic anemias. Because data on the prevalence of these 5 residual causes are limited, their impacts on anemia are estimated by first removing the impact of all other known causes of anemia and then assigning the remaining cases to these 5 residual causes at fixed proportions. In other words, the prevalence of dietary iron deficiency anemia is estimated as a proportion of the counterfactual anemia (i.e., the proportion of anemia in the absence of other known anemia causes). Direct incorporation of iron status data (which do not differentiate by cause of iron deficiency) is not readily possible within the GBD cause framework because, as described, this framework requires the disease burden to be assigned directly to underlying causes.

#### Estimate of anemia burden due to dietary iron deficiency

Disability weights, which represent the extent of health loss associated with a specific health outcome, are used to calculate YLDs. Specifically, the disability weight (95% CI) assigned for mild anemia is 0.004 (0.001, 0.008), for moderate anemia is 0.052 (0.034, 0.076), and for severe anemia is 0.149 (0.101, 0.210) (57). This method captures only the direct burden of anemia due to dietary iron deficiency. The indirect effects of iron deficiency on other conditions, such as maternal mortality, low birth weight, or impaired physical performance, for example, would be considered in the analysis of iron deficiency as a risk factor.

### Estimate of disease burden due to iron deficiency in the GBD 2019 Study, using the risk factor method

#### Estimate of exposure to iron deficiency

As described, for the risk factor method in GBD 2019, iron deficiency as a risk factor includes all anemia subtypes that can manifest as iron deficiency and would therefore have the potential to respond to iron supplementation. The prevalence of iron deficiency is quantified in terms of hemoglobin concentration. The exposure to iron deficiency is estimated by removing all of the anemia subtypes that are not related to iron deficiency (Supplemental Table 1), leaving all of those that are iron-related in a single group. The latter disorders include causes of excessive blood loss, as well as conditions that impair iron absorption (e.g., maternal hemorrhage, uterine fibroids, menstrual disorders, hookworm, schistosomiasis, gastritis and duodenitis, inflammatory bowel disease). This was achieved in GBD 2019 by summing the combined prevalence times hemoglobin shift from each of the conditions categorized as not being iron-related and adding that sum to the observed mean hemoglobin (from the anemia envelope ST-GPR model). The theoretical minimum risk exposure level (TMREL), as the “normal” hemoglobin by age, sex, and pregnancy status, was estimated as the 95^th^ percentile of mean hemoglobin concentration across all GBD location-years.

#### Estimate of disease burden risk-attributable to iron deficiency

For GBD 2019, only 2 causes of anemia were identified as having sufficient evidence to support burden attribution to iron deficiency. One cause was dietary iron deficiency, where the attributable fraction was assigned 100% to iron deficiency. The second cause was maternal disorders and all subcauses of maternal disorders. PAFs for linking maternal disorders to iron deficiency were calculated in the standard way by combining exposure estimates with corresponding outcome-specific RRs. RRs of iron deficiency were derived from the meta-analyses by Murray-Kolb et al. (69, 70), which found an RR (95% CI) for all-cause maternal mortality of 1.252 (1.087, 1.425). This study did not assess iron status, iron deficiency, or iron supplementation, but rather assessed hemoglobin concentration as a risk factor for overall (i.e., not cause-specific) maternal mortality and the underlying sources could not be identified to facilitate updating meta-analysis using MR-BRT. An “analogy” criterion was used to assign hemoglobin-derived RR information to iron deficiency, which was facilitated by expressing the exposure of iron deficiency in terms of iron-related hemoglobin decrement rather than prevalence of iron deficiency. In GBD 2016, only 2 of 9 subcauses of maternal disorders—maternal hemorrhage and maternal sepsis and other maternal infections—were estimated as having burden attributable to iron deficiency. In GBD 2017, this decision was revisited during annual collaborator consultation sessions and revised to include all maternal disorders subcauses in recognition of the evidence from the meta-analyses by Murray-Kolb et al. (69) only being for all-cause maternal mortality. The same RR from Murray-Kolb et al. was applied for all maternal subcauses and the PAFs for each were therefore equivalent (58). This procedure resulted in GBD 2017 and GBD 2019 having higher estimates of the burden attributable to iron deficiency than previous GBD studies.

### Critique of methods used for iron-related estimates

The use of hemoglobin concentration as a proxy to estimate the prevalence of iron deficiency is discouraged because of concerns about the assumptions related to how much of the anemia is due to iron deficiency in different settings, as recently confirmed by the Biomarkers Reflecting Inflammation and Nutritional Determinants of Anemia (BRINDA) project using results from national surveys with iron biomarker data (71, 72). Such variation in the iron-related proportion of anemia is reflected in the GBD 2019 estimates, but the concordance between GBD and BRINDA findings has yet to be evaluated quantitatively. An additional challenge that the GBD Collaboration faces is that many available hemoglobin results derive from surveys conducted in low- and middle-income countries (14). Although the surveys conducted between 1990 and the present include assessments in most population groups, they focus primarily on the most vulnerable groups, such as preschool children (*n *= 400 surveys), pregnant women (*n *= 290 surveys), and women of reproductive age (*n *= 251 surveys), with fewer surveys among other population groups such as adolescents (*n *= 152 surveys), men (*n *= 144 surveys), school-age children (*n *= 114 surveys), and elders (*n *= 64 surveys). Thus, the data used to create anemia prevalence estimates for all age groups by sex and by country and over time require statistical models to fill in data gaps. Then, in the second step of causal distribution, the GBD Collaboration estimates the proportion of anemia due to dietary iron deficiency, which relies on assigning all estimated cases of anemia to a single cause using the hemoglobin shift of every known cause of anemia, the proportion of residual cases attributed to each residual cause, and the full distribution of hemoglobin concentration. Much of this information is sparse, and conceptually the possibility that anemia causes often overlap is ignored. In other words, this method does not allow for anemia due to multiple causes, such as combined iron deficiency and chronic or recurrent infectious diseases. It also does not consider the health consequences of iron deficiency without anemia, leading to likely underestimation of the full disease burden of iron deficiency. The biggest limitation is the sparsity of reliable iron status data. Data collection on various age groups and in additional locations is urgently needed to fill gaps empirically and facilitate widespread validation and improvement of statistical models of hemoglobin, anemia, and iron deficiency.

## Estimate of Disease Burden Due to Zinc Deficiency in the 2019 GBD Study, Using the Risk Factor Method

### Estimate of prevalence of zinc deficiency

The estimate of the disease burden due to zinc deficiency only uses the risk factor method, and is applied only to children 1–4 y of age. Because of the limited amount of representative information available on plasma zinc concentration from different populations, the prevalence of zinc deficiency is estimated based on dietary intake data from nationally and subnationally representative nutrition surveys and on food availability data obtained from FAO SUAs (after adjusting for food waste). This information is then used to predict the mean zinc intake at the population level, and to characterize the distribution of zinc intake, as a proxy for zinc status (58, 73). Zinc deficiency was defined as consumption of <2.5 mg Zn/d, which is the estimated average requirement (EAR) for children 1–4 y of age based on the US Institute of Medicine (74). The analyses do not account for phytase content in foods, because a model based on isotopic tracers of zinc absorption among young children found no detectable effect of phytate (75). When assessing zinc intake, the GBD Collaboration considered 24-h dietary recall surveys as the gold standard and FAO SUA data were corrected for bias using MR-BRT. This Bayesian meta-regression model uses covariate data to try to adjust for differences between studies (e.g., demographics of the study population) and prior distributions to aid the estimation of model parameters (62). MR-BRT is used for cross-walks (i.e., mappings between ≥2 standards) and bias adjustments. That is, MR-BRT was used to estimate and correct for systematic differences in the FAO data sources compared with what would be expected from 24-h recall data (58). The GBD Collaboration used their ST-GPR model followed by ensemble distribution fitting (a similar approach to that described already for anemia estimation) to estimate the mean intake of zinc by age, sex, country, and year. To assist with prediction for locations and years without data, the GBD Collaboration also used the lag-distributed income and energy availability (kcal) of that location-year as a covariate in GBD 2019 (58).

### Estimate of disease burden due to zinc deficiency

For the GBD 2019 Study, the GBD Collaboration performed their own meta-analyses of randomized controlled trials of zinc supplementation among young children using MR-BRT. This is a departure from the GBD 2017 Study, where the RRs of selected illnesses due to zinc deficiency were obtained by pooling the RRs from studies included in the most recently published meta-analyses by Mayo-Wilson et al. (4). The meta-analyses performed for the GBD 2019 Study have not yet been published, thus details will be described here briefly. The use of MR-BRT resulted in the removal of lower respiratory tract infections, and the RR (95% CI) for diarrhea were updated to 0.88 (0.83, 0.93). These differences are due to the studies included in the meta-analyses and the methods used to determine the RRs. Briefly, the GBD 2019 included 5 additional trials not considered in the Mayo-Wilson meta-analyses (76–80). Further, Mayo-Wilson et al. (4) had calculated the RRs for each trial, whereas the GBD 2019 used the study-reported RRs when available and the Mayo-Wilson RRs otherwise. In the GBD 2019 Study, the RRs were not adjusted for the country-specific prevalence of zinc deficiency due to the lack of a significant relation between the background prevalence of deficiency (defined on the basis of the dietary and SUA data) and the magnitude of the RR.

### Critique of methods used for zinc-related estimates

Estimating the prevalence of zinc deficiency based on dietary availability from the national food supply likely underestimates the true prevalence of zinc deficiency, as has been found in countries with nationally representative data on plasma zinc concentration (81). An additional concern is that there are presently 2 global dietary databases available, and results from each lead to different conclusions regarding nutrient adequacy (82). Moreover, serum or plasma zinc concentration is controlled by a homeostatic mechanism and responds only slightly to changes in dietary zinc intake (32, 83). Thus, although zinc availability in the food supply may be useful to identify the potential risk of zinc deficiency for a specific country, it is not recommended as a proxy for the prevalence of zinc deficiency. Nevertheless, opinions differ on acceptable methods to estimate the prevalence of a micronutrient deficiency when data are scarce, as discussed during a technical consultation jointly organized by the WHO and the US CDC (84). Participants at that consultation reportedly agreed that a conceptual framework may be useful to help understand underlying factors and processes that are expected to influence a prevalence estimate and that such a framework requires flexibility to allow for inclusion of biological, behavioral, or health care system covariates at the study and country levels. Concerns were raised that there is a risk of making an analytical model increasingly complex and that, even if covariates conceptually make sense, the quality of the data across countries and data availability may limit the usefulness of the covariates (84).

## Estimate of Disease Burden Due to Vitamin A Deficiency in the GBD 2019 Study

The estimates of the disease burden due to vitamin A deficiency rely on both the causal attribution method and the risk factor method. The same approach to estimate the prevalence of vitamin A deficiency is used for both methods, as we will describe.

### Estimate of prevalence of vitamin A deficiency

The GBD Collaboration estimated the prevalence of vitamin A deficiency using a 3-step process. In the first step, the prevalence of vitamin A supplementation coverage was estimated using an ST-GPR model (e.g., timing of the introduction of a supplementation program). In the second step, estimates of high-dose vitamin A supplementation were used as a covariate in the ST-GPR model for the prevalence of vitamin A deficiency. This model used serum retinol concentrations < 0.7 µmol/L for age- and sex-specific groups using WHO VMINIS (14) as the primary data source to assign the prevalence of vitamin A deficiency, with some additional data from DHSs and other surveys. Before these data were analyzed, they were cross-walked to create sex-specific and age-specific estimates of deficiency prevalence from combined sex and age estimates using MR-BRT and DisMod-MR, respectively. The covariates used in the model were the estimated vitamin A supplementation coverage (as described), sociodemographic index, age-specific stunting SEV, the log of lag-distributed income per capita, and availability of retinol activity equivalent units in the national food supply. In the third step, the prevalence of vision loss due to vitamin A deficiency is estimated, as we will describe.

### Estimate of disease burden due to vitamin A deficiency, using the causal attribution method

The prevalence of vision loss due to vitamin A deficiency was estimated using DisMod-MR with the estimated vitamin A deficiency prevalence as the only covariate. The case of vision loss due to vitamin A deficiency is defined as the presence of a corneal scar, which is in line with the definition used in the WHO VIMNS database (14). The input data for this model were split by sex using the sex ratio for vision loss due to vitamin A deficiency (estimated using a separate MR-BRT model) (57). The following assumptions were made in the modeling procedure: no excess mortality, possibility of birth prevalence, and reduction in incidence and remission of vitamin A deficiency after 5 y of age. Consequently, the incidence estimates were driven by the age pattern of prevalence after allowing for remission. The total vision loss is then cross-walked into moderate vision loss, severe vision loss, and blindness using DisMod-MR using age, socio-demographic index, and an index of health care access and quality (57). The disability weight (95% CI) assigned for moderate vision loss is 0.031 (0.019, 0.049), for severe vision loss is 0.184 (0.125, 0.258), and for blindness is 0.187 (0.124, 0.260) (57).

### Estimate of disease burden due to vitamin A deficiency, using the risk factor method

For the GBD 2019 Study, the GBD Collaboration reanalyzed results from randomized controlled trials of vitamin A supplementation among young children using MR-BRT. Similarly to the methods used for zinc, the most recently published meta-analyses by Imdad et al. (3) calculated the RRs for each trial, whereas GBD 2019 used the study-reported RRs when available and the Imdad et al. RRs otherwise. The result of this analysis was that lower respiratory tract infections were not statistically significant, and thus were removed as an outcome of vitamin A deficiency, and the RRs for diarrhea and measles were updated (decreased in both cases). In GBD 2019, the RRs were not adjusted for the country-specific prevalence of vitamin A deficiency due to lack of a significant relation between the magnitude of the RR and the background prevalence of deficiency.

### Critique of methods used for vitamin A-related estimates

Vitamin A deficiency is the only micronutrient included in the GBD Study presently based on the recommended biomarker and deficiency cutoff. For the GBD 2019 Study, the GBD Collaboration ran their own meta-analyses using MR-BRT. The conclusion was that vitamin A supplementation is no longer significantly associated with lower respiratory tract infection, which is in line with conclusions of the most recently published meta-analyses by Imdad et al. (3). In contrast, the RR (95% CI) derived for vitamin A supplementation on measles indicated a smaller benefit in the GBD meta-analysis (RR: 0.72; 0.53, 0.97) than in the findings by Imdad et al. (RR: 0.50; 0.37, 0.67). This is partly due to the exclusion of 3 studies which only had seroconversion as the primary outcome (85–87). The rationale for excluding these studies was that they did not report on the RR for the incidence or mortality of measles and a health burden is the outcome of interest in the GBD Study. These meta-analyses have not yet been published independently by the GBD Collaboration, and such a publication will be important to understand what assumptions were made.

## Discussion

Direct assessment of the prevalence of micronutrient deficiencies and their related disease burdens requires measurement of known biomarkers of micronutrient status among representative samples of the population subgroups of interest. Because relevant information is inadequate for most micronutrients of likely public health concern, alternative methods have been applied to estimate the prevalence of these deficiencies. Among the 4 micronutrients included in the GBD 2019 Study, only vitamin A deficiency is presently based on the recommended biomarker and deficiency cutoff. For iodine, iron, and zinc deficiencies, proxy indicators are used, which leads to a discrepancy between the definitions generally used in the field of nutrition and those used in the GBD Study (**Table 4**) and different, and possibly inaccurate, prevalence estimates being published by different groups. Moreover, the use of different data sources and analytical methods limits comparability across micronutrients and between micronutrients and other risk factors for disease outcomes.

**TABLE 4:** Terminology and definitions of population-level micronutrient deficiency status commonly used in the field of nutrition research compared with the GBD Study^1^

Terminology	Nutritionists’ definition	Summary of indicators and modeling approach used in the GBD Study
Iodine deficiency	Concentrations of urinary iodine or thyroglobulin below or above the cutoff, respectively; prevalence of total goiter (palpable, but not visible; and visible) (34–36)	Visible goiter
Anemia	Hemoglobin concentration cutoff specific to each population subgroup (as defined by age, sex, and physiological status) (67)	Hemoglobin concentration below the population-specific cutoff (67)
Dietary iron deficiency	Usual intake of iron from diet less than the population-specific EAR, as determined by dietary assessments, such as 24-h recalls	Risk exposure for dietary iron deficiency modeled based on hemoglobin concentration below the anemia cutoff after accounting for other known anemia causes, and after accounting for known causes of iron deficiency (such as hookworm, schistosomiasis, upper gastrointestinal bleeding, and gynecologic conditions)
Iron deficiency	Concentration of iron status biomarkers below (i.e., ferritin) or above (i.e., transferrin receptor, zinc protoporphyrin) the cutoff (38, 39); indicators commonly adjusted for indicators of inflammation (i.e., C-reactive protein, α-1-acid glycoprotein) (47, 48)	Risk exposure for iron deficiency modeled based on hemoglobin concentration below the anemia cutoff after accounting for other known anemia causes
Zinc deficiency	Concentration of plasma zinc below the population-specific cutoff (41); plasma zinc commonly adjusted for indicators of inflammation (i.e., C-reactive protein, α-1-acid glycoprotein), especially in young children (49)	Dietary zinc inadequacy estimated from dietary surveys and FAO SUAs, especially in young children
Vitamin A deficiency	Concentration of retinol or retinol-binding protein below the cutoff (45); concentrations often adjusted for indicators of inflammation (i.e., C-reactive protein, α-1-acid glycoprotein) (50)	Concentration of serum retinol below the cutoff

1 EAR, estimated average requirement; GBD, Global Burden of Disease; SUA, Supply Utilization Account.

The methods used to estimate the prevalence and disease burden differ for each of the 4 micronutrients in the GBD Study, and some of the methods have changed with new iterations of the GBD Study. The changes over time reflect the approach taken by the GBD Collaboration to continuously update the GBD Study and are the reason why each GBD Study supersedes the previously published version. In some cases, these changes may be due to newly available data or scientific evidence. In other instances, the changes may be due to newly available statistical models, such as MR-BRT, which was developed to deal with problematic assumptions inherent in other meta-regression methods (62). For example, for the GBD 2017 Study (88), the disease burdens due to vitamin A and zinc deficiency were estimated by pooling the RRs from studies included in published meta-analyses (3, 4), and the GBD Collaboration performed the meta-analyses with the metafor package in R (89). In contrast, the GBD 2019 Study used the reported RRs of the individual randomized controlled trials, if available, and the GBD Collaboration performed the meta-analyses using MR-BRT (58). As a result of multiple methodological changes, the estimated disease burdens for these 2 micronutrients were substantially lower in the GBD 2019 Study than in previous GBD Studies(90). These examples highlight that the GBD Study is undergoing continual development. Although the inclusion of newly available data and scientific evidence and the development of new models are important for improving global health metrics, such as the GBD estimates, some of these changes may alter previous conclusions and trigger uncertainty about the accuracy of the estimates.

The present review focused on the assessment of iodine, iron, zinc, and vitamin A because these are included in the GBD Study, which served as the case study for this review. Several other micronutrients, however, have public health consequences. Specifically, a recent initiative to develop a strategic plan to increase the availability and utilization of reliable data on population micronutrient status globally also emphasized the need for more data on folate, vitamin B-12, vitamin D, and thiamin (6, 7), whose deficiencies may result in physical disability, sensory impairments, restricted physical growth, impaired neurocognitive development, or death (21–23, 91–93). Other micronutrients such as riboflavin, niacin, and pyridoxine and mineral elements such as calcium and selenium may be equally important for public health, but information about them is even more limited because of the scarcity of population status data (7). Without population-level assessments of these micronutrient deficiencies, we will remain uncertain about their public health impact and thus fail to address and prevent potential deficiencies and associated health consequences. Thus, assessing biomarkers of more micronutrients in nationally or regionally representative surveys is urgently needed to shed light on the extent of the problem.

Because the available data for micronutrient deficiency are sparse for many locations, statistical modeling is required in many facets of estimating the global burden of disease. For micronutrient deficiencies, many locations have sparse data and prevalence estimates above or below a certain threshold can have a major impact on the perceived need for, or success of, an intervention. Transparency and validation of the modeling assumptions and requirements are critical so that the limitations of the analyses can be assessed by experts in the field. The presentation of valid uncertainty intervals (i.e., intervals with an appropriate amount of uncertainty) is a critical aspect of reporting results. The uncertainty of GBD input data includes combined uncertainty from both sampling and nonsampling variance. The GBD Collaboration then applies Bayesian methods to model each quantity, routinely using out-of-sample predictive validity testing when feasible (i.e., when data sets are sufficiently large), and samples 1000 draws from the posterior distribution of each model. Uncertainty is propagated through subsequent calculations by randomly combining sets of 1000 draws so as to avoid any assumption about correlation (e.g., uncertainty in one country is independent of uncertainty in another). In theory this approach should produce valid uncertainty intervals, but complex multistep approaches have uncertainty estimates that can be sensitive to modeling assumptions and risk underestimating uncertainty (94). Appropriate reporting of uncertainty can also point to major data gaps and can indicate where more resources should be allocated to data generation. The importance of transparency has been recognized by many (95), and the GBD Study adheres to the Guidelines for Accurate and Transparent Health Estimates Reporting (GATHER) (96). Transparency requires producers of global burden estimates to have clear documentation, testing, and validation of assumptions and valid reporting of uncertainty, which will help the consumers of global burden estimates understand the complexity of the process and the importance and value of the uncertainty reported with the results. In addition, Shiffman and Shawar (27) suggested that organizations involved in global health metrics should design and disseminate their work cautiously and with potential adverse effects in mind.

Because of the present scarcity of information, micronutrient deficiencies remain largely hidden and overlooked, resulting in insufficient progress toward achieving the 2030 SDG agenda (97, 98). This is particularly relevant because addressing micronutrient deficiencies is among the most cost-efficient intervention strategies to improve health (99, 100). Moreover, relying on proxy indicators to predict the health burden of risk factors such as micronutrient deficiencies, even with quantification of valid uncertainty intervals, may obscure the data sparseness and leave the impression that we know more than we do, which can lead to a lack of investments in relevant data collection.

## Conclusions

Estimating the disease burden due to micronutrient deficiencies is a continuous and iterative process and requires harnessing new data sources and new data processing methods as they become available. At present, the lack of micronutrient status data from representative surveys is a major limitation in the attempt to estimate the global prevalence of micronutrient deficiencies and related disease burden, highlighting the need to collect more micronutrient status data from nationally representative surveys. Because of these limitations in data availability, complex statistical modeling is required to produce current estimates using assumptions and proxies that likely understate the true extent of deficiencies and the consequent global health burden.

## Supplementary Material

nzab141_Supplemental_File

## Notes

Supported in part by Bill & Melinda Gates Foundation grant INV-003021 to the Micronutrient Forum (to SJMO). Under the grant conditions of the Foundation, a Creative Commons Attribution 4.0 Generic License has already been assigned to the Author Accepted Manuscript version that might arise from this submission.

Author disclosures: RR works for the Bill & Melinda Gates Foundation. KHB, the spouse of SYH, works as a consultant for the Micronutrient Forum. All other authors report no conflicts of interest.

The funder had no role in the present review, except for RR, an employee of the Bill & Melinda Gates Foundation, who contributed to editing the manuscript.

Supplemental Table 1 is available from the “Supplementary data” link in the online posting of the article and from the same link in the online table of contents at https://academic.oup.com/cdn/.

SYH and ACM contributed equally to this work.

Abbreviations used: BRINDA, Biomarkers Reflecting Inflammation and Nutritional Determinants of Anemia; DALY, disability-adjusted life-years; DHS, Demographic Health Survey; DisMod-MR, disease modeling—meta regression; FBS, food balance sheet; GBD, Global Burden of Disease; MR-BRT, meta regression—Bayesian, regularized, trimmed; PAF, population-attributable fraction; SDG, Sustainable Developmental Goal; SEV, summary exposure value; ST-GPR, Spatio-Temporal Gaussian Process Regression; SUA, Supply Utilization Account; VMNIS, Vitamin and Mineral Nutrition Information System; YLD, years of life lived with disability; YLL, years of life lost.
