# Supplementary material for: Challenges for Estimating the Global Prevalence of Micronutrient Deficiencies and Related Disease Burden: A Case Study of the Global Burden of Disease Study
Source: Curr Dev Nutr. 2021 Nov 18;5(12):nzab141. doi: 10.1093/cdn/nzab141 (PMC8728001; doi:10.1093/cdn/nzab141)
Supplement: nzab141_Supplemental_File [file nzab141_supplemental_file.docx]

**On-line Supplementary Material**

**Challenges for estimating the global prevalence of micronutrient deficiencies and related disease burden: A case study of the Global Burden of Disease Study**

Sonja Y. Hess, Alexander C. McLain, Edward A. Frongillo, Ashkan Afshin, Nicholas J. Kassebaum, Saskia J. M. Osendarp, Reed Atkin, Rahul Rawat^,^ Kenneth H. Brown

**Supplementary Table 1:** Anemia subtypes in the GBD 2019 study

| **Causes with prevalence and hemoglobin shift inputs** | **Considered iron-related in risk factor model** |
| --- | --- |
| *P. falciparum* parasitemia without clinical malaria |  |
| *P. vivax* parasitemia without clinical malaria |  |
| Clinical malaria |  |
| Schistosomiasis | Iron-related |
| Hookworm disease | Iron-related |
| Maternal hemorrhage | Iron-related |
| Vitamin A deficiency | Iron-related |
| Peptic ulcer disease | Iron-related |
| Gastritis | Iron-related |
| Stage III chronic kidney disease | Iron-related |
| Stage IV chronic kidney disease | Iron-related |
| Stage V chronic kidney disease | Iron-related |
| End stage renal disease | Iron-related |
| Uterine fibroids | Iron-related |
| Menstrual disorders | Iron-related |
| G6PD deficiency |  |
| Hemizygous G6PD deficiency |  |
| Beta-thalassemia major |  |
| Beta-thalassemia trait |  |
| Hemoglobin E trait |  |
| Hemoglobin E/beta-thalassemia |  |
| Hemoglobin H disease |  |
| Homozygous sickle cell and severe sickle cell/beta-thalassemia parent |  |
| Hemoglobin SC disease |  |
| Mild sickle cell/beta-thalassemia |  |
| Sickle cell trait |  |
| HIV | Iron-related |
| Cirrhosis and other chronic liver diseases, decompensated | Iron-related |
| Ulcerative colitis | Iron-related |
| Crohn’s disease | Iron-related |
| **Estimated via Fixed Proportion Redistribution Methods^1^ in GBD Anemia Causal Attribution** |  |
| Dietary iron deficiency | Iron-related |
| Other infectious diseases | Iron-related |
| Other neglected tropical diseases | Iron-related |
| Other endocrine, nutrition, blood, and immune disorders | Iron-related |
| Other hemoglobinopathies and hemolytic anemias |  |

G6PD, Glucose-6-phosphate dehydrogenase

**^1^**A minimum of 10% of all anemia is assigned to residual categories based on analysis of NHANES-III data from the United States
